# Two cases of hemodynamic improvement by modulation of atrioventricular delay in cardiac operations

**DOI:** 10.1186/s40981-022-00546-z

**Published:** 2022-07-27

**Authors:** Akiko Tomita-Kobayashi, Tomoko Fujimoto, Shoko Takada, Yuki Masuda, Akinobu Mizutani, Yukio Hayashi

**Affiliations:** 1 Anesthesiology Service, Sakurabashi-Watanabe Hospital, Osaka, Japan; 2 Present address: Anesthesiology Service, Osaka City General Hospital, 2-13-22 Miyakojima-hondori, Miyakojima-ku, Osaka, 531-0021 Japan; 3 Radiologic Technology Service, Sakurabashi-Watanabe Hospital, Osaka, Japan

**Keywords:** Cardiac pacemaker, Sick sinus syndrome, Pacing mode, Cardiac operation

## Abstract

**Background:**

Symptomatic sick sinus syndrome is one of the indications for pacemaker implantation, and we have to consider to program the pacemaker to an asynchronous pacing mode during an operation.

**Case presentation:**

We reported two cases with a pacemaker implanted for sick sinus syndrome undergoing cardiac operation. We changed programming of the pacemaker to an asynchronous pacing mode (DOO) and modulated the programmed atrioventricular delay to avoid ventricular pacing, resulting in better hemodynamic condition. Although we observed premature ventricular contraction, no lethal arrhythmias induced by the R-on-T phenomenon were noted.

**Conclusion:**

Programming of the pacemaker to an asynchronous pacing mode and modulation of the programmed atrioventricular delay to avoid ventricular pacing may be an option for pacemaker management during an operation.

## Background

Symptomatic sick sinus syndrome (SSS) is one of the indications for pacemaker (PM) implantation. Dual-chamber rate-modulated (DDD) pacing may be a desirable pacing mode in SSS patients with impaired atrioventricular (AV) conduction or concern over future [1]. One of the most important questions is how to manage the patient with a PM during an operation. It is generally accepted to program the pacemaker to an asynchronous pacing mode, such as VOO or DOO, if the patient is pacemaker-dependent. On the other hand, when a patient’s heart rate is sometimes greater than the dual-chamber pacing rate, to increase the programmed rate of the PM over the patient’s heart rate by programming the PM to an asynchronous pacing mode may be an option during an operative procedure.

Hemodynamic stability is one of the important issues during anesthetic management of patients undergoing a cardiac operation. Now, we reported two cases with a PM implanted for SSS undergoing a cardiac operation. We changed to program the PM to an asynchronous pacing mode (DOO) and modulated the programmed AV delay to avoid ventricular pacing, leading to better hemodynamic status.

## Case presentations

Written patient consent was obtained, and our institutional ethical committee approved the publication of this report.

### Case 1

A 77-year-old female (153 cm, 60 kg) was scheduled to undergo mitral valve replacement (MVR) and tricuspid annuloplasty (TAP) for severe mitral regurgitation (MR) and moderate tricuspid regurgitation (TR). Her past history included aortic valve replacement for severe aortic stenosis and pacemaker implantation for SSS. Her preoperative cardiac function was as follows: left ventricular ejection fraction (LVEF) 58%, ischemia in the apex, left ventricular end-diastolic and end-systolic diameter (LVDd/Ds) 50/35 mm, left atrial diameter 40 mm, no aortic regurgitation, severe MR with mitral annular enlargement and valve degeneration, and moderate TR with tricuspid annular enlargement and TR pressure gradient 18 mmHg. The preoperative pacemaker programming mode was DDD with a programmed rate of 60 bpm, and the electrocardiogram (ECG) showed all atrial-pacing with QT prolongation (QTc = 0.526 s). However, there was no evidence of atrioventricular block, premature atrial contractions (PACs), or premature ventricular contractions (PVCs).

**Fig. 1:**
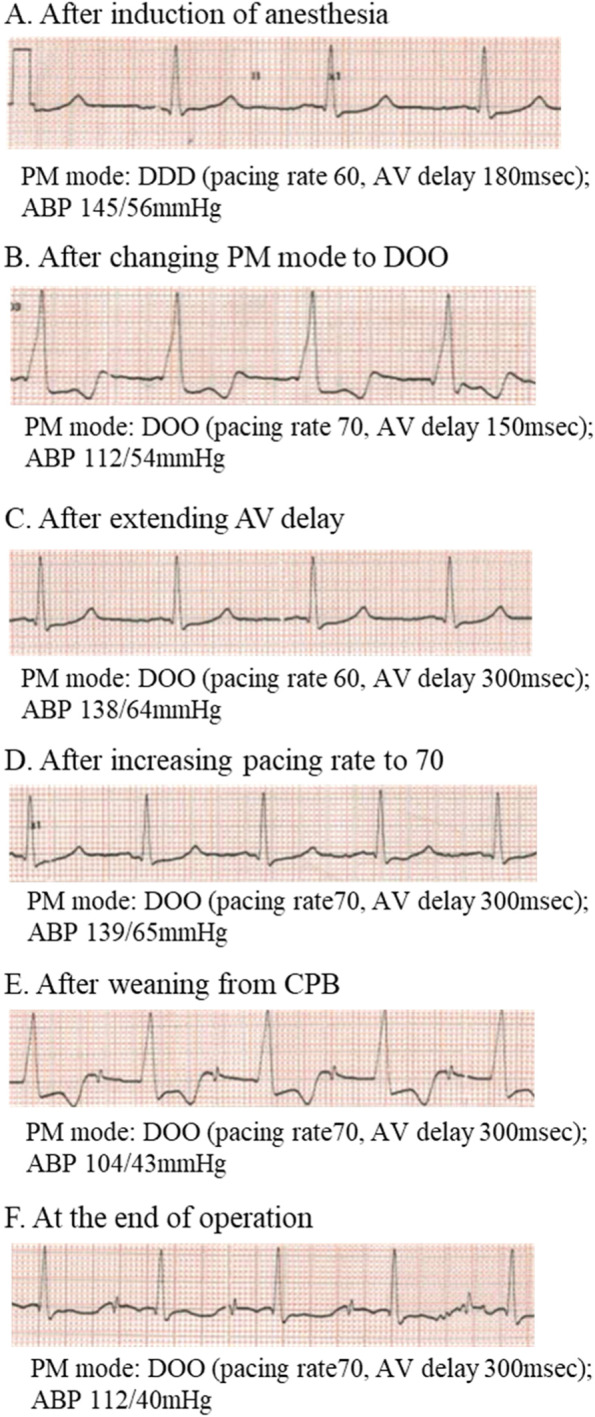
Electrocardiographic changes at each indicated point (**A**–**F**) during anesthesia in case 1. The pacing mode and arterial blood pressure at each point are shown. The QRS width changes from wide to narrow by adjusting the atrioventricular delay (**B**–**D**). Ventricular pacing is activated due to atrioventricular block immediately after weaning from the cardiopulmonary bypass (**E**), but atrioventricular conduction improves at the end of the operation (**F**). PM pacemaker, ABP arterial blood pressure, AV atrioventricular, CPB cardiopulmonary bypass

Anesthesia was induced with midazolam 5 mg, fentanyl 0.2 mg, and vecuronium 8 mg, and maintained with sevoflurane, propofol, remifentanil, and vecuronium. After induction of general anesthesia, the pacemaker programming was changed to DOO. In addition, we increased a programmed rate to 70 bpm. As expected, the ECG waveform showed all atrial-ventricular-pacing with a wide QRS waveform. However, her systolic blood pressure decreased by 20 mmHg (Fig. 1A, B). The original programmed AV delay was 150 ms, and the patient’s own PR interval had been mildly prolonged to 154 ms, so we changed the programmed rate to 60 bpm and extended the AV delay to 300 ms. With these settings, the wide QRS waveform changed to a narrow one, and the arterial blood pressure increased (Fig. 1C). Then, while we increased the programmed rate to 70 bpm, the narrow QRS waveform and arterial blood pressure were maintained (Fig. 1D). After changing the programing, we observed the patient’s ECG for a while to confirm that no arrhythmias occurred. Thus, the operation was started with this pacing program, and cardiopulmonary bypass was established without any problem. Immediately after weaning from CPB, the ECG showed an all ventricular-pacing waveform with AV block in the presence of dobutamine 4 μg^.^kg^−1.^min^−1^ and noradrenaline 0.01 μg^.^kg^−1.^min^−1^ (Fig. 1E). However, the atrioventricular conduction improved with time, although the catecholamines administered were decreased to dobutamine 3 μg^.^kg^−1.^min^−1^ alone. The ECG waveform at the end of the operation was almost similar to that before cardiopulmonary bypass (Fig. 1F). The operation was completed without any problems. After the surgery, the pacemaker setting was returned to the original mode.

### Case 2

A 72-year-old female (167 cm, 69 kg) was scheduled to undergo MVR and TAP for severe MR and moderate TR. Her past history included mitral valvuloplasty for severe MR and pacemaker implantation for SSS. The preoperative cardiac function was as follows LVEF 71%, LVDd/Ds 63/37 mm, left atrial diameter 60 mm, severe MR with postmedial mitral annular ring dissociation and moderate TR with tricuspid annular enlargement, and TR pressure gradient 36 mmHg. The preoperative pacemaker programming mode was DDD with a programmed rate of 50 bpm. The preoperative ECG showed sinus rhythm with a heart rate of 64 bpm and sporadic PVCs. The pacemaker records showed 25% atrial-pacing and no ventricular-pacing. There was no AV block, PAC, or QT prolongation.

**Fig. 2:**
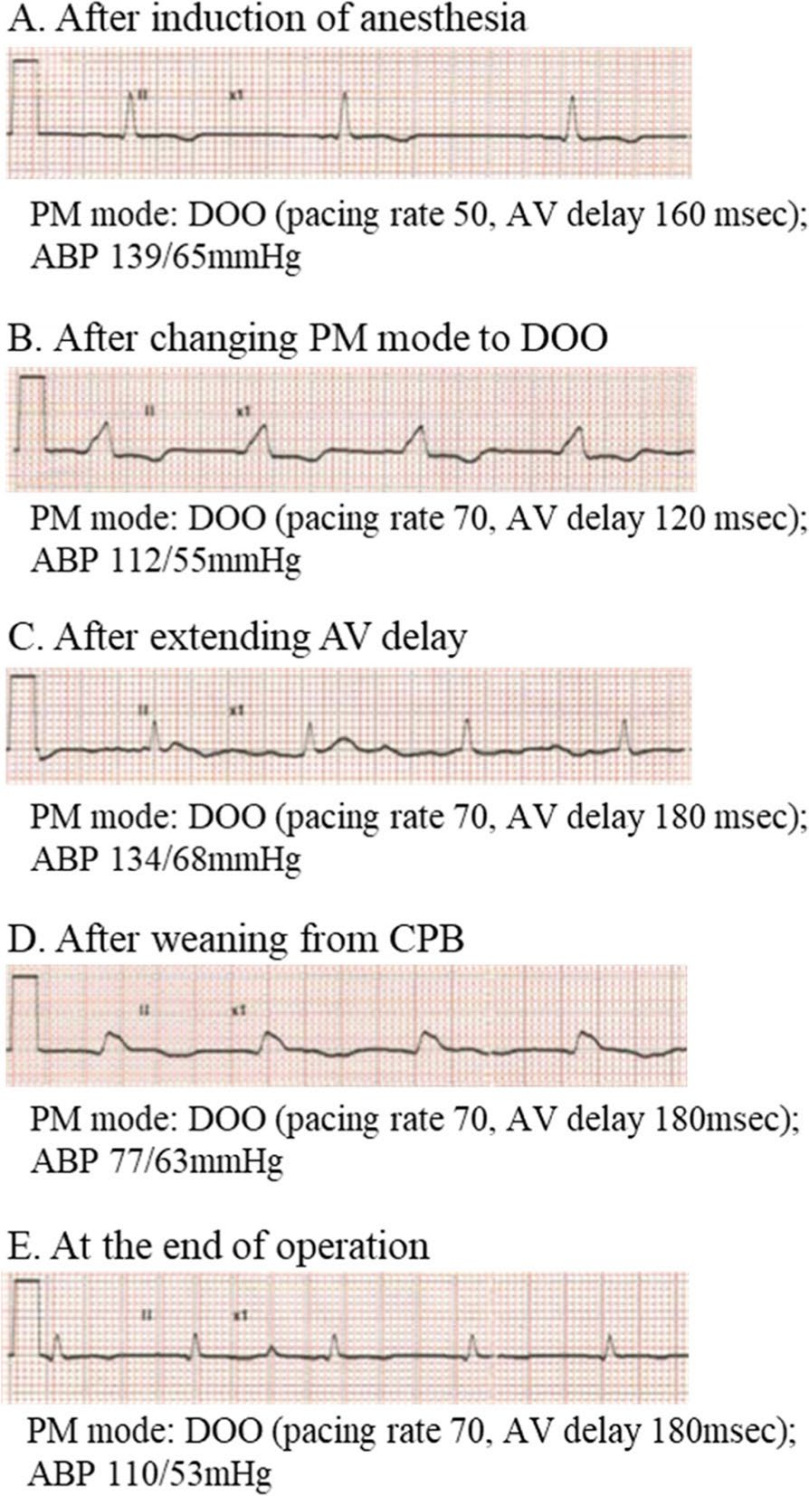
Electrocardiographic changes at each indicated point (**A**–**E**) during anesthesia in case 2. The pacing mode and arterial blood pressure at each point are shown. The QRS width changes from wide to narrow by adjusting the atrioventricular delay (**B**–**C**). Ventricular pacing is activated due to atrioventricular block immediately after weaning from the cardiopulmonary bypass (**D**), but atrioventricular conduction improves at the end of the operation (**E**). PM pacemaker, ABP arterial blood pressure, AV atrioventricular, CPB cardiopulmonary bypass

Anesthesia was induced with midazolam 5 mg, fentanyl 0.2 mg, and vecuronium 8 mg, and maintained with sevoflurane, propofol, remifentanil, and vecuronium. After induction of general anesthesia, as in case 1, the pacemaker programming was changed to DOO with a programmed rate of 70 bpm and a programmed PR interval of 120 ms, resulting in a wide QRS waveform with all atrial-ventricular-pacing (Fig. 2A, B). Thus, we increased the AV interval to 180 ms, which was slightly longer than her PR interval, leading to the timing of ventricular pacing to be comparable to the patient’s intrinsic ventricular activation, even if the ventricular activation occurred by the PM. We considered that this setting may reduce the risk of R on T. Then, we could see a narrow QRS instead of a wide one associated with an increase in blood pressure (Fig. 2B, C). After changing the programming, we confirmed that no arrhythmias occurred. The operation was started, and cardiopulmonary bypass was established without any problems. Immediately after weaning from CPB, the ECG showed an all ventricular-pacing waveform with AV block in the presence of dobutamine 4 μg^.^kg^−1.^min^−1^ and noradrenaline 0.01 μg^.^kg^−1.^min^−1^ (Fig. 2D). However, the atrioventricular conduction improved with time and the QRS waveform at the end of the operation became narrow (Fig. 2E) in the presence of dobutamine 4 μg^.^kg^−1.^min^−1^. Although sporadic PVCs were observed during the operation, no lethal arrhythmias occurred, and the operation was completed without any problems. After the surgery, the pacemaker setting was returned to the original mode.

## Discussion

It is not rare to change programming of the PM to asynchronous activity during an operation to prevent inhibition while electrocautery is being used. In the present cases, we changed to program the PM to an asynchronous pacing mode (DOO) and modulated the programmed AV delay to avoid ventricular pacing and to obtain a better hemodynamic status in patients with a PM implanted for SSS undergoing cardiac operation.

The previous study by Nitardy et al. [2] documented that dual-chamber rate-modulated pacing with a long AV delay is effective to reduce unnecessary right ventricular pacing and the guideline for PM recommended a significance of minimizing unnecessary ventricular pacing with a long AV delay [1]. We applied this idea to the anesthetic management of the present cases, resulting in better hemodynamic status.

In the first case, the patient’s PR interval was slightly prolonged to 154 ms, and the original programmed AV delay was shorter, so all atrial-ventricular-pacing was programmed in the DOO mode (Fig. 1B). Thus, we set maximally programmable AV delay in the DOO mode to maintain normal ventricular activation, resulting in a narrow QRS and blood pressure elevation (Fig. 1B, C). However, one may be afraid of the possibility that ventricular pacing remains because of the DOO mode, and this stimulation may have a risk of spike on T. However, this may be unlikely. We increased the programmed rate to 70 bpm to suppress the patient’s atrial activation completely and ventricular stimulation by the PM with the programmed AV delay was within the absolute refractory period of the ventricle in the presence of QT prolongation [3]. In fact, after changing the program, we watched the patient’s ECG for a while, and no arrhythmias were observed.

In the second case, PM management was similar to that of case 1 except for the shorter AV delay because the second patient had a normal QT interval, and we had to reduce the risk of the R-on-T phenomenon by sporadic PVCs when the PM was programmed to an asynchronous pacing mode [4, 5]. Nevertheless, we have to acknowledge that we cannot avoid this possibility completely, when we use an asynchronous pacing mode. However, the patient underwent an open heart operation and cardiac surgeons are familiar with electrical cardioversion; thus, this situation may be further justification for our management plan in patient 2.

One may claim that it was unnecessary to change the PM to an asynchronous pacing mode in patient 2, because his preoperative pacemaker records showed 25% atrial-pacing and no ventricular-pacing, suggesting that this patient was not completely pacemaker-dependent. Maintaining DDD mode is definitely useful to prevent the R-on-T phenomenon. However, there was concern about the negative effect of anesthetics on the sinus node and PM failure during the operation. If a patient with PM who is not completely PM-dependent, as in case 2, undergoes non-cardiac surgery, we might not change the PM to an asynchronous pacing mode. As described above, we think that our management plan might be justified for open heart procedures.

## Conclusion

We reported two cases with PMs implanted for SSS undergoing cardiac operation. We changed to program the PM to an asynchronous pacing mode (DOO) and modulated the programmed AV delay to avoid ventricular pacing, resulting in better hemodynamic status.

## Data Availability

The data that support the findings of this report are available from the corresponding author on reasonable request.
