# Correction: Two cases of hemodynamic improvement by modulation of atrioventricular delay in cardiac operations

**DOI:** 10.1186/s40981-022-00561-0

**Published:** 2022-08-27

**Authors:** Akiko Tomita-Kobayashi, Tomoko Fujimoto, Shoko Takada, Yuki Masuda, Akinobu Mizutani, Yukio Hayashi

**Affiliations:** 1 Anesthesiology Service, Sakurabashi-Watanabe Hospital, Osaka, Japan; 2 Present address: Anesthesiology Service, Osaka City General Hospital, 2-13-22 Miyakojima-hondori, Miyakojima-ku, Osaka, 531-0021 Japan; 3 Radiologic Technology Service, Sakurabashi-Watanabe Hospital, Osaka, Japan

**Correction: JA Clin Rep 8, 56 (2022)**

**https://doi.org/10.1186/s40981-022-00546-z**

Following the publication of the original article [1], the author reported that “Anesthesiology Service, Sakurabashi-Watanabe Hospital, Osaka, Japan” was missing.

This has been corrected in this correction article and the original article has been updated.
